# Allelopathic Effects of Native Versus Invasive Plants on One Major Invader

**DOI:** 10.3389/fpls.2019.00854

**Published:** 2019-07-02

**Authors:** Gabrielle Thiébaut, Michèle Tarayre, Héctor Rodríguez-Pérez

**Affiliations:** ECOBIO, UMR 6553 CNRS, Université de Rennes 1, Rennes, France

**Keywords:** plant-plant interactions, invasive species, *Ludwigia hexapetala*, *Ludwigia peploides*, *Myriophyllum aquaticum*, *Mentha aquatica*

## Abstract

Allelopathy is defined as the effects (stimulatory and inhibitory) of a plant on the development of neighboring plants through the release of secondary compounds. Autoallelophaty is the beneficial or harmful effect of a plant species on itself. The allelopathic potential belonging to a native species could induce a biotic resistance against invasive plants, whereas allelochemicals released by exotic species could favor the establishment of invasive species (invasional meltdown). The aim of our study was to examine the potential allelopathic effect of four plant species on the target species *Ludwigia hexapetala* using two experiments. In the first experiment, we tested the allelopathic effect of root and leaf leachates of the two congeneric exotic species *Ludwigia hexapetala* and *Ludwigia peploides* on *L. hexapetala,* while in the second experiment, we studied the allelopathic effect of root and leaf leachates of a sympatric exotic species *Myriophyllum aquaticum* and of one native species *Mentha aquatica* on *L. hexapetala*. We measured the stem length to calculate the relative growth rate and four physiological traits (nitrogen balance index and flavonol, chorophyll, anthocyanin indices) of the target plants on a weekly basis. At the end of the experiment, we determined the aboveground and belowground biomass. We also counted the number of lateral branches and measured their lengths. We found that the root leachates of *L. peploides* and of *Myriophyllum aquaticum* had stimulated the synthesis of flavonols of *L. hexapetala.* Leaf leachate of *L. hexapetala* also stimulated its own flavonol synthesis. Also, the root leachate of *L. peploides* had stimulated the total biomass and length of lateral branches of *L. hexapetala,* whereas the production of lateral branches had been stimulated by root leachates of both *Ludwigia* species and by leaf leachate of *Myriophyllum aquaticum.* The autoallelopathy of *L. hexapetala* could explain its invasiveness. Both leachates produced by *Mentha aquatica* had no effect on the physiological and morphological traits of the invasive *L. hexapetala* and indicated no biotic resistance in the recipient community. The two invasive plant species *Myriophyllum aquaticum* and *L. peploides* could favor the establishment of *L. hexapetala*. These results suggested an “invasional meltdown.”

## Introduction

Many aquatic plant species have been introduced to other continents either accidentally or voluntary for ornamental purposes for example. An invasive species is one that spreads outside their natural range and may impact the native diversity and overall structure and function of ecosystems. Invasive species often establish monospecific patches in their introduced ranges but coexist with neighbors in their native habitat (Ridenour and Callaway, 2001). Many studies suggest that allelopathy may contribute to the ability of exotic species to form dense stands in invaded ecosystems (Hierro and Callaway, 2003). Allelopathy, defined as the chemical interactions between plants or plants and microorganisms, could have either positive or negative effects on the performance of neighbors (Rice, 1984). Apart from affecting the establishment of coexisting species, allelopathic species can also affect their own establishment and self-regeneration. When the target plant is also the donor, the phenomenon is called autoallelopathy, which is a type of intraspecific interaction. Few studies showed a positive effect of autoallelopathy on the growth of the plant itself (Zhu et al., 2015; Bardon et al., 2017). The suspicion that allelochemicals, released by a root or leaf, may interfere with neighbors has been the subject of different theories on biological invasions such as the “novel weapon hypothesis” (NWH, Callaway and Aschehoug, 2000; Callaway and Ridenour, 2004) or the “Biotic Resistance” hypothesis (Elton, 1958). However, many ecosystems often contain combinations of exotic species. These communities of invaders could be driven by facilitation or mutualistic interactions between exotic species, according to the theory of “invasional meltdown” (Simberloff and Von Holle, 1999). An invasional meltdown is defined by the interactions which leads one invasive species to favor the invasion of one or more other exotic species. Moreover, plants that have co-evolved with a species with an allelopathic ability may be less susceptible to allelochemicals, while newly exposed species may exhibit less resistance (theory NWH, Callaway and Aschehoug, 2000). Consequently, allelochemicals released by native plants could also affect the growth of invasive species and would, thus, constitute a biotic resistance against plant invasion (Christina et al., 2015).

However, some exotic species may limit the establishment of other exotic species. Indeed, exotic species that have not co-evolved with the invasive one could be sensitive to allelochemichals (Callaway and Aschehoug, 2000), whereas sympatric species could be favored by secondary compounds released by the donor species (Ehlers and Thompson, 2004). Moreover, the secondary metabolite composition of plants is phylogenetically determined (Grutters et al., 2017) and two close species may produce similar chemical compounds. Consequently, two conspecific species are less susceptible to allelochemicals released by their own individuals and by those of the other species. The allelopathic effects of native plants on exotic plants and on the interactions between conspecific and heterospecific invasive plants have rarely been studied.

This paper is focused on a major invader in wetlands, the water primrose *Ludwigia hexapetala* (Hook. and Arn.) (syn. *L. grandiflora* subsp. *hexapetala*). Native to South America, this species was introduced into France in 1830 and spread initially within the Mediterranean region of France and later into Europe (Thouvenot et al., 2013). The water primrose has been listed on the European List of Invasive Exotic Species since July 2016. The rapid and extensive development of *Ludwigia* spp. populations can block waterways, irrigation ditches, and canals; impact human activities (navigation, hunting, fishing, irrigation, and drainage); reduce biodiversity (Stiers et al., 2011); and degrade water quality (Thouvenot et al., 2013). *L. hexapetala* is a perennial aquatic plant which forms very dense mats. It grows horizontally in water (or mud) and can break the water surface. It is mainly aquatic, but is also able to colonize terrestrial habitats such as riverbanks and wet meadows (Thouvenot et al., 2013). The terrestrial form of *L. hexapetala* has recently invaded wet meadows along the Atlantic Coast of France leading to the depreciation of the fodder value of meadows, resulting in the abandonment of pasture (Billet et al., 2018). In a previous study, we established that *L. hexapatala* stimulated its own germination and could promote its own population persistence (Santonja et al., 2018). *L. hexapetala* produces allelochemicals (Dandelot et al., 2008; Santonja et al., 2018; Thiébaut et al., 2018) and these substances could be implicated in the outcome of the interactions between the water primrose and the surrounding species, be they native or exotic.

The aim of our study was to test whether individuals of *L. hexapetala* would modulate their morphological and physiological traits after exposure to the leachate of different species. The morphological traits were related to plants’ ability to grow, to regenerate, and to colonize new habitats. The physiological traits were indicative of plant’s allocation of resources to growth or to defenses, the plant’s ability to photosynthesize, and an indicator of an exposure to stress. In other words, we investigated the autoallelopathy of *L. hexapetala* and whether a native species and two sympatric species have the ability to promote or to inhibit the establishment of the water primrose through allelopathy. The first hypothesis was that the leachate of the congeneric plant *Ludwigia peploides* (Kunth) Raven ssp. *montevidensis* (Spreng.) Raven have no negative effect on the performance of *L. hexapetala*, because these two species have a common historic exposure to the allelochemicals (theory NWH) and that the leachates of *L. hexapetala* promote the growth of itself. The second hypothesis was that the putative allelochemicals released by the native species *Mentha aquatica* L. have a negative effect on the growth of the invasive species *L. hexapetala* (Biotic Resistance Hypothesis), whereas the putative secondary compounds produced by the sympatric species *Myriophyllum aquaticum* (Vell.) Verdc have a positive effect on *L. hexapetala* (invasional meltdown theory).

## Materials and Methods

### Donor Species

Native to South America, *L. peploides* (primrose-willow) was imported into France around 1830 from the South East as an ornamental plant (Dutartre, 2004). It is now a widespread species and has been listed on the European List of Invasive Exotic Species since July 2016. *L. peploides* often forms monospecific stands and outcompetes other aquatic species (Dutartre, 2004). It is a creeping emergent macrophyte. It can root in the substrate and send out long prostrates or ascending stems that freely root and branch at nodes and often create dense mats. Dandelot et al. (2008) showed that *L. peploides* possess an allelopathic activity that induces a seedling chlorosis, a decrease in germination and an increase in mortality for watercress *Nasturtium officinalis* R. Br.

Originating in South America, the creeping emergent Parrot’s Feather *Myriophyllum aquaticum* was introduced into Europe, more specifically, into France, in the 1880s (Sheppard et al., 2006). It was imported for use in aquaria and garden ponds but escaped into the wild. The European Union has banned the sale and planting or keeping of this plant, even in isolated ponds. Regulations are met for *Myriophyllum aquaticum*. The plant’s stems may float out over the surface to form dense stands, from which the emergent shoots rise, making impenetrable mats (Hussner, 2009). Once introduced into a new region, the plants easily spread downstream mainly in the form of vegetative fragments. It is often found in eutrophic waters (small water bodies, irrigation channel networks, and small streams). This species has demonstrated a potential inhibitory effect on neighboring plants (Elakovich and Wooten, 1989). Saito et al. (1989) showed a significant inhibitory activity on growth of the blue-green algae *Microcystis aeruginosa* f. *aeruginosa* (strain number NIES-44) and *Anabaena flos-aquae* f. *flos-aquae* (NIES-73).

*Mentha aquatica* is a perennial plant from the northern temperate regions of Europe. It has a creeping rhizome with submerged leaves and the erect stems possess aerial leaves. *Mentha aquatica* is typically associated with permanently wet habitats adjacent to open water, often partially or wholly submerged. The invasive *L. hexapetala* and the native *Mentha aquatica* can co-occur in the wild in European aquatic ecosystems. The watermint *Mentha aquatica* is recognized as having an allelopathic effect (Santonja et al., 2018).

### Experimental Design

Two experiments were conducted with the target species *L. hexapetala*. In the experiment 1, the two donor species were the two congeneric species *L. hexapetala* and *L. peploides,* whereas in the experiment 2, the donor species were *Myriophyllum aquaticum* and *Mentha aquatica*. In the experiment 1, the aim was also to test the potential autoallelopathy of *L. hexapetala* on its growth.

#### Experiment 1

In mid-May 2018, 50 shoots of *L. hexapetala* were collected from Apigné pond (01°44′25.2″W, 48°05′41.4″N). For each shoot, an apical shoot (hereafter called individual), without buds or lateral stems, was cut to a length of 8 cm. In the laboratory, the individuals were washed to remove invertebrates, algae, and debris. They were acclimatized for 2 weeks in deionized tap water at room temperature (19°C). They float free in deionized water. The individuals produced roots during this acclimatization period. After these 2 weeks, each individual was planted in a pot (7 cm in diameter and 8 cm in height), containing 50% fertile agricultural soil (NPK = 14:10:18 kg/m^3^, pH = 6) and 50% of sand.

Leaves and roots of *L. hexapetala* and of *L. peploides* were selected in the spring of 2018 from the Apigné pond in Brittany for *L. hexapetala* and the Brière Marshland (02°26′41″W, 47°32′63″N) for *L. peploides*. Only, the small, round, floating leaves (i.e., those in contact with water) were collected. The leaves were detached from the stems, washed to remove benthic invertebrates and filamentous algae, and stored in the dark at 4°C. Leaf and root leachates of *L. hexapetala* and *L. peploides* were separately prepared by soaking 10 g of fresh leaves and 10 g of fresh roots (equivalent dry weight) in 1,000 ml of deionized water for 12 h in darkness. These 1% aqueous solutions were then filtered through a filter paper (Whatman #1). The leaf and root leachates were then stored at 4°C for 24 h prior to the experiment.

At the start of the experiment, each individual of the target species *L. hexapetala* was watered either with 15 ml of deionized water for the control (C) or with 15 ml of a leaf/root leachate (1%) of the donor species, either *L. peploides* or *L. hexapetala.* Target individuals were watered with leaf or root leachate only once at the beginning of the experiment. Each treatment and the control had 10 replicates. Pots were randomly positioned in a growth chamber (Photon Flux Density 100 μmols^−1^ m^2^, 14 h light/10 h dark cycle) at 21°C for 28 days. The bottom of the pots was kept in tap water (*ca*. 1–2 cm depth). Individuals of the target species *L. hexapetala* were watered with deionized water to maintain by to keep the substrate wet, once each week for 4 weeks.

##### Measurement of Morphological and Physiological Traits

We used a functional trait approach to study the responses of individuals of *L. hexapetala* after an exposure to root and leaf leachates. We measured both physiological and morphological traits. Four physiological traits were measured simultaneously *in vivo* using a non-destructive measurement device called the Dualex Scientific+™ sensor. This is a hand-held leaf-clip sensor (Cerovic et al., 2012; Bürling et al., 2013) that measures flavonols (Flav.), anthocyanin (Anth.), and chlorophyll (Chl.) indices and calculates the nitrogen balance index (NBI). The NBI is more of an indicator of C/N allocation changes due to N deficiency (from 0 to 100) than a measure of leaf nitrogen content *per se*. The Chl. index related to the chlorophyll content (between 0 and 150) is an indicator of the photosynthetic yield. The Flav. index related to the flavonol content or to phenolics accumulation is an indicator of the defense mechanisms against pathogens and herbivores. The Anth. index related to anthocyanin is an indicator of an exposure to stress (shading conditions, nutrient deficiencies, temperature stress etc.). The measurements of physiological traits were taken from two apical leaves per individual of *L. hexapetala* per pot. We repeated these physiological measurements on five individuals (i.e., 10 measurements per week).

Five morphological traits were measured. We counted lateral branches and measured their length (Barrat-Segretain et al., 1998). Based on the number of roots, we evaluated plant ability to colonize. To obtain a proxy of the apical growth, we measured stem length and then calculated the relative growth rate (RGR; d − 1) according to Hunt (1990):

$$RGR\;\mathrm{stem}=\left(\ln\;L2–\ln\;L1\right)/\left(T2–T1\right)$$

where L1 and L2 represent total shoot length, at the beginning (T1) and end of the experiment (T2), respectively.

At the end of the experiment, the main shoot length and the lateral shoot length were measured, the lateral branches were counted, and the roots and shoots were harvested. The above and belowground vegetative parts of the plants were dried separately at 65°C for 72 h and weighed. The ratio of belowground to aboveground mass was calculated.

#### Experiment 2

Leaves and roots of the two donor species *Mentha aquatica* and *Myriophyllum aquaticum* were collected once more in mid-June of 2018 from two different ponds in Apigné in Brittany, France. We also collected 50 individuals of the target species *L. hexapetala.* At these sampling sites, the two donor and the target species did not co-occur. The leaf and root leachates of each species (*Mentha aquatica, Myriophyllum aquaticum*) were prepared according to the protocol followed in Experiment 1. The same experimental design was applied and the same morphological and physiological traits were measured as in Experiment 1. The duration of this experiment was 3 weeks.

### Data Analysis

Longitudinal data in both experiments, RGR, and the physiological parameters NBI, chlorophyll, flavonols, and anthocyanin were analyzed on the basis of repeated measures. Whenever data met parametric test assumptions, a linear mixed model with a split-plot design for repeated measures was applied, including each plant as a random factor; otherwise data were analyzed by means of non-parametric testing (Naguchi et al., 2012). We double checked that data met the parametric test assumptions graphically and by means of the Shapiro-Wilk test (residuals normality) and Breusch-Pagan test (homoscedasticity test). A type III ANOVA model was used to test hypothesis in parametric repeated measures analyses to RGR data in both experiments and NBI, chlorophyll, and flavonols in Experiment 2. Whenever the interaction between treatment effect and sampling time was significant, a *post hoc* pairwise comparison was performed among treatments for each sampling period individually. We used *t*-test comparisons for parametric datasets and Mann-Whitney-Wilcoxon test for non-parametric datasets; in both cases, *p’s* were subsequently corrected by means of Benjamini-Hochberg False Discovery Rate test for multiple comparisons with a 10% acceptance level (Benjamini and Yekutieli, 2001).

The effects of the leachate source upon total mass and root/stem mass ratio were tested with a one-way ANOVA test, the model II, owing to an imbalance among the number of plants in the treatments in the two experiments. The root/stem mass ratio was in each occasion transformed with logit transformation to improve normality and homoscedasticity of residuals. Total biomass log transformation was needed only for the *L. hexapetala* dataset in the first experiment. In both experiments, the effects of the leachate origin upon the number of branches, including plants without branches in the datasets, were analyzed with a generalized linear model following a Poisson error distribution. The effect of the leachate origin upon the total length of branches was tested with a one-way ANOVA, type II, excluding from the analyzed dataset the plants without branches. All analyses were performed with R software (R Core Team, 2016).

## Results

### Effects of Root and Leaf Leachates Of *L. Hexapetala* and of *L. Peploides* on *L. Hexapetala* (Experiment 1)

**Figure 1:**
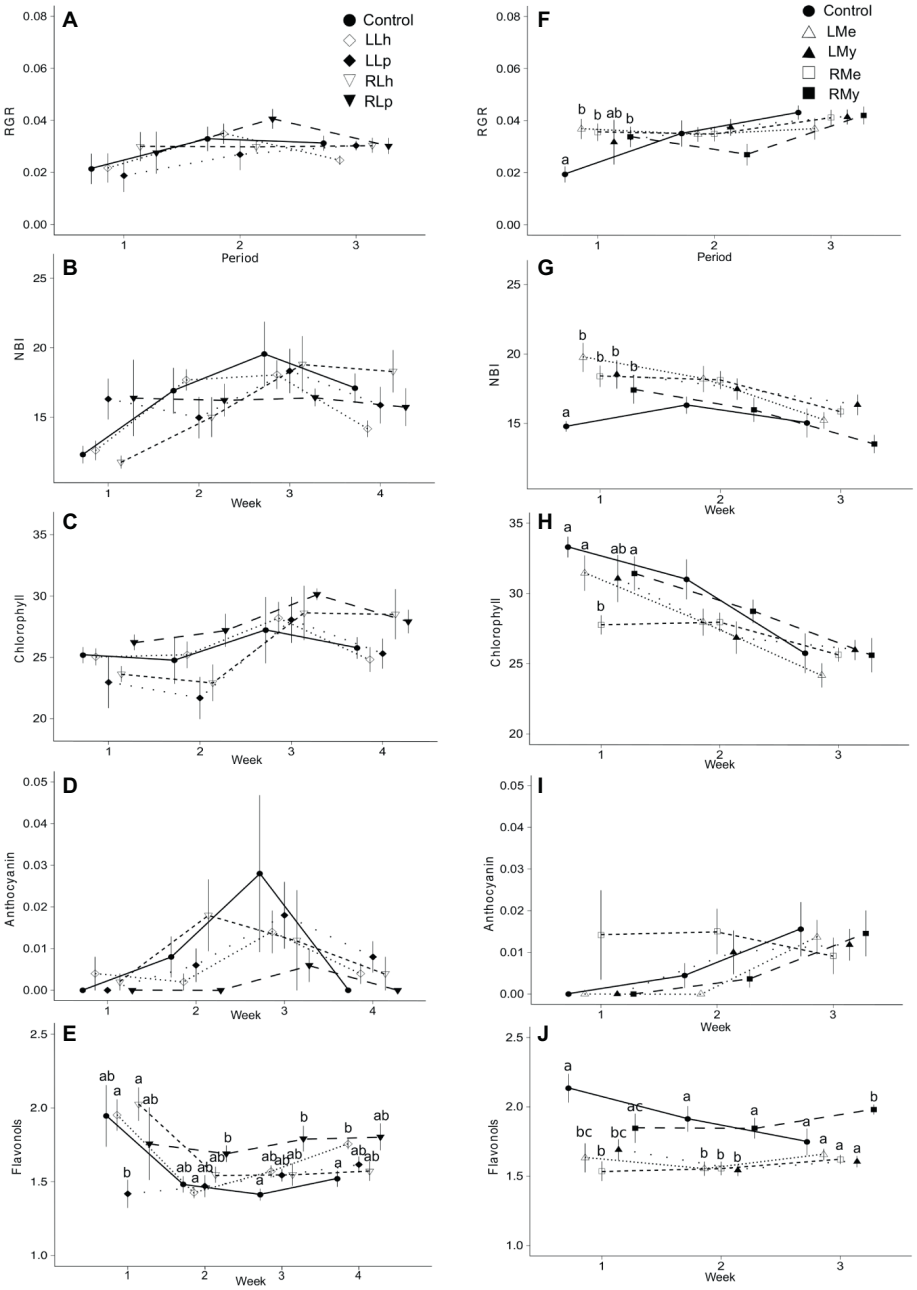
Mean values plus standard error of longitudinal data from experiments 1 and 2. **(A–E)** show the effects of root and leaf leachates of *L. hexapetala* and *L. peploides* plants upon *L. hexapetala* plants, and **(F–J)** show the effects of root and leaf leachates of *Myriophyllum aquaticum* and *Mentha aquatica* upon *L. hexapetala* plants. RGR refers to relative growth rate assessed between two consecutive sampling times. In **(A–E)**, solid black circles = control values, white rotated squares = *L. hexapetala* leaf leachate (LLh), solid black rotated squares = *L. peploides* leaf leachate (LLp), white inverted triangles = *L. hexapetala* root leachate (RLh), solid black inverted triangles = *L. peploides* root leachate (RLp). In **(F–J)**, solid black circles = control values, white triangles = *Mentha aquatica* leaf leachate (LMe), solid black triangles = *Myriophyllum aquaticum* leaf leachate (LMy), white squares = *Mentha aquatica* root leachate (RMe), solid black square = *Myriophyllum aquaticum* root leachate (RMy). Letters set the significance of pairwise comparisons (significance threshold of 0.05).

The interaction of two factors (time and leachate origin) was significant for the synthesis of flavonols in leaves of *L. hexapetala* after 3 and 4 weeks, respectively (*p* = 0.01, Figure 1E, Table 1). The root leachate of *L. peploides* and the leaf leachate of *L. hexapetala* significantly stimulated the synthesis of flavonols of *L. hexapetala* after 3 and 4 weeks, respectively. There was no significant effect of both leaf and root leachates of both *L. hexapetala* and *L. peploides* on the NBI, chlorophyll, and anthocyanin indices (Figures 1A–D, Table 1).

**Table 1:** Effects of leaf/root leachates on physiological traits of *L. hexapetala* (experiment 1: leachates of *L. peploides* or *L. hexapetala*; experiment 2: leachates of *Myriophyllum aquaticum* or *Mentha aquatica*).

Physiological traits		Experiment 1			Experiment 2		
		Statistic	df	*p*	Statistic	df	*p*
RGR	Treatment	4.93	4	0.4	13.30	4	**0.009**
	Time	2.46	1	0.1	21.16	1	**<0.0001**
	Treatment × Time	2.40	4	0.7	11.60	4	**0.02**
NBI	Treatment	0.16	3.08	0.9	18.49	4	**<0.0001**
	Time	0.92	2.41	**<0.0001**	0.05	1	0.8
	Treatment × Time	1.89	6.40	0.07	11.48	4	**0.02**
Chl	Treatment	2.44	3.37	0.06	5.86	4	**0.003**
	Time	10.49	2.61	**<0.0001**	23.48	1	**<0.0001**
	Treatment × Time	0.74	6.86	0.6	9.68	4	**0.05**
Flav	Treatment	4.16	3.40	**0.004**	42.11	4	**<0.0001**
	Time	7.35	1.96	**0.007**	18.75	1	**<0.0001**
	Treatment × Time	2.92	5.47	**0.01**	23.22	4	**0.0001**
Anth	Treatment	1.36	2.93	0.3	1.77	3.62	0.1
	Time	6.25	2.47	**0.0008**	18.39	1.63	**<0.0001**
	Treatment × Time	1.44	6.52	0.2	1.70	5.31	0.1

*Longitudinal data analysis results based on repeated measures analysis. RGR, relative growth rate; NBI, nitrogen balance index; Chl, chlorophyll index; Flav, flavonol index; Anth, anthocyanin index. Significant *p*’s at 5% significance level are in bold*.

**Figure 2:**
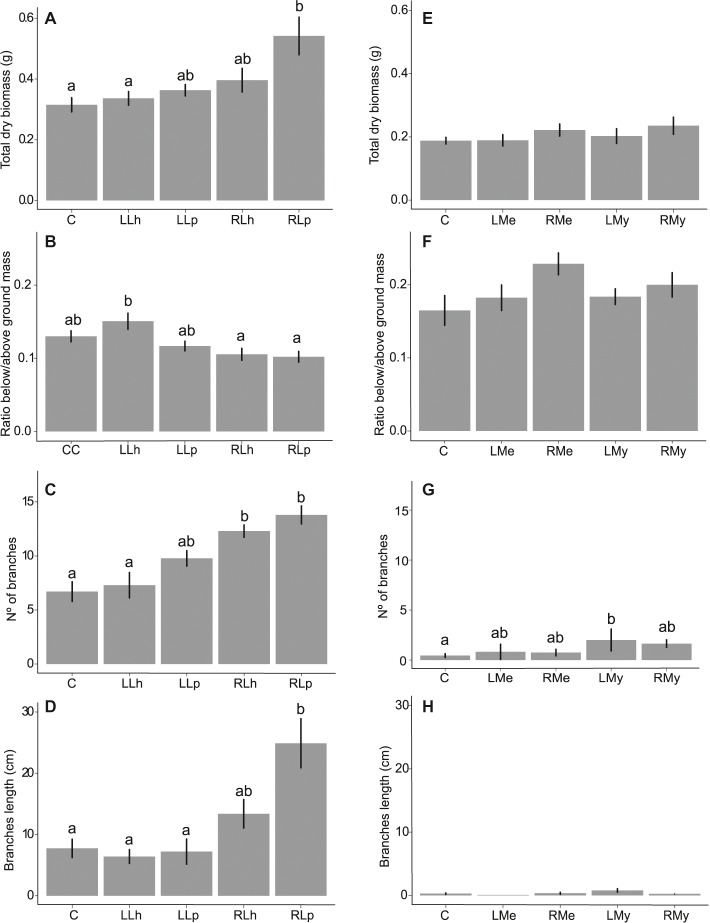
Mean values plus standard error of morphological traits data from Experiment 1 **(A–D)** and 2 **(E–H)**. Leachate treatments are represented in the abscissa axis, where control is always identified by **(C)**. In **(A–D)**, *L. hexapetala* leaf leachate is identified by LLh, *L. peploides* leaf leachate by LLp, *L. hexapetala* root leachate by RLh, and *L. peploides* root leachate by RLp. In **(E–H)**, *Mentha aquatica* leaf leachate is identified by LMe, *M. aquatcum* leaf leachate by LMy, *Mentha aquatica* root leachate is identified by RMe, and *Myriophyllum aquaticum* root leachate by RMy. Letters set the significance of pairwise comparisons (significance threshold of 0.05).

There was no effect of the leaf and root leachates of *L. hexapetala* and *L. peploides* on the RGR of *L. hexapetala* (Table 1). All the morphological traits were significantly influenced by the type of leachate (Table 2). The total biomass of *L. hexapetala* was stimulated by the root leachate of *L. peploides (F* = 4.80; *p* = 0.003, Figure 2A). The ratio below/aboveground mass was not significantly affected by the leachates (Figure 2B). The number of branches of *L. hexapetala* was stimulated both by the root leachate of *L. hexapetala* (Chi = 36.93; *p* < 0.0001) and *L. peploides* (Chi = 32.97; *p* < 0.0001, Figure 2C). The lengths of lateral shoots of *L. hexapetala* were longer after exposure to the root leachate of *L. peploides (F* = 4.69; *p* = 0.003, Figure 2D). There was no effect of leaf leachates of both *Ludwigia* species on the morphological traits of *L. hexapetala* (Figure 2).

**Table 2:** Effects of leaf/root leachates on morphological traits of *L. hexapetala* observed at the end of the experiment (experiment 1: leachates of *L. peploides* or *L. hexapetala*; experiment 2: leachates of *Myriophyllum aquaticum* or *Mentha aquatica*).

Morphological traits	Experiment 1			Experiment 2		
	Statistic	df	*p*	Statistic	df	*p*
Total biomass	4.05	4	**0.003**	0.84	4	0.5
Below/aboveground mass ratio	4.15	4	**0.006**	2.04	4	0.1
Lateral branches length	3.47	4	**0.02**	1.63	4	0.2
Number of branches	36.93	4	**<0.0001**	14.30	4	**0.006**

*Significant *p’s* at 5% significance level are in bold*.

The root leachate of *L. hexapetala* did not affect its own physiological traits, apical growth, biomass, and branching (Figure 2).

### Effects of Root and Leaf Leachates Of *Mentha aquatica* and *Myriophyllum aquaticum* on *L. Hexapetala* (Experiment 2)

The interaction of two factors (time and leachate origin) was significant for NBI and the chlorophyll content in *L. hexapetala* at the beginning of the experiment (Figure 1F, Table 1). The individuals watered with deionized water (control) were characterized during the first week by a lower RGR and NBI than the individuals watered by root or leaf leachates of *Mentha aquatica* or *Myriophyllum aquaticum* (Figures 1F,G, Table 1). The chlorophyll content in control was higher than that in the individuals exposed to root leachates of *Mentha aquatica* during the first week of the experiment (Figures 1H,J). There was no effect of root and leaf leachates of *M aquatica* and *Myriophyllum aquaticum* on the anthocyanin synthesis of *L. hexapetala* (Figure 1I, Table 1). The interaction of two factors (time and leachate origin) was significant for the synthesis of flavonols of *L. hexapetala (p* = 0.0001, Figure 1J, Table 1). After 3 weeks, root leachate of *Myriophyllum aquaticum* stimulated the synthesis of flavonols in *L. hexapetala* (Figure 1J).

There was no effect of leaf and root leachates of *Mentha aquatica* on the morphological and physiological traits of the target species (Figures 2E–H, Table 2). There was effect of leaf and root leachates of *Myriophyllum aquaticum* on the total dry biomass, on ratio below/aboveground mass and on the length of branches (Figures 2E,F,H, Table 2). The number of branches of *L. hexapetala* was stimulated by the leaf leachate of *Myriophyllum aquaticum* (Figure 2G).

## Discussion

### Allelopathic Effects of Leaf and Root Leachates of *L. Peploides* and Of *L. Hexapetala* on the Traits of *L. Hexapetala* (Experiment 1)

The leachates of *L. peploides* and of *L. hexapetala* affected the physiological and morphological traits of *L. hexapetala*. Leachates from leaves of *L. hexapetala* and from roots of *L. peploides* stimulated the flavonol synthesis of *L. hexapetala*. The increase of flavonols content in the epidermis of *L. hexapetala* is a surrogate of leaf dry mass per area. The epidermis of the leaves of all plants contains flavonoids that protect against UV-B radiation (280–320 nm). These compounds absorb light in the UV-B range but allow visible light to pass through uninterrupted for photosynthesis and consequently enhanced photosynthesis. Flavonoids act as signal molecules to take preventive measures against attack and were consequently implied in the mechanisms of defenses (Samanta et al., 2011). They increased in *L. hexapetala*, whereas no apical growth (RGR) was established. The allocation of energy to defense versus growth (“The dilemma of plant,” Herms and Mattson, 1992) is particularly important for the invasive plant persistence in field. Harms et al. (2017) reported strong herbivory damage in *L. hexapetala* in spring. However, despite the diverse assemblage of herbivores and fungi associated with *L. hexapetala*, damage was relatively low and the plant continues to persist as an invasive species (Harms et al., 2017). The root leachates of both *Ludwigia* spp. increasing the synthesis of flavonols contributed to the resistance of *L. hexapetala* to herbivores. This represents an efficient strategy for *L. hexapetala*.

The ability of both *Ludwigia* spp. to release allelochemicals by roots into the soil may increase nutrient availability (Bardon et al., 2017; Thiébaut et al., 2018) and consequently stimulate the branching of *L. hexapetala*. More specifically, the lateral growth (number and length of lateral branches) and the total biomass of *L. hexapetala* were enhanced in the presence of the root leachate of *L. peploides*. The stimulation of branching and biomass increased the vigor, the regeneration, and the colonization abilities of *L. hexapetala*. These positive effects of root leachate of *L. peploides* could be considered as facilitation interactions and “invasional meltdown.” Though rare, literature data established that facilitation among congeneric plants does occur and is referred to as “intraspecific” facilitation (Loayza et al., 2017). The strong allelopathic potential of two *Ludwigia* species leads to think that water-soluble compounds released from these plants play a significant role in the successful invasion of these aquatic macrophytes. A similar result was reported with two invasive aquatic plants of *Alternanthera* species (Abbas et al., 2016). Our first hypothesis on facilitation effect of congeneric species *L. peploides* on the growth of *L. hexapetala* was validated.

We also found a positive autoallelopathy of *L. hexapetala* leachates on the flavonols synthesis and on the production of lateral shoots. These results are congruent with a previous work in which leaf leachates of *L. hexapetala* have been reported to stimulate seed germination of itself (Santonja et al., 2018). Similarly, Zhu et al. (2015) showed that root extracts of *Ailanthus altissima* stimulated seed germination, elongation of radicle extension, and elongation of seedlings of itself. Few examples of positive effects of autoallelopathy on plant’s growth were reported (Zhu et al., 2015; Bardon et al., 2017) and on their implication to the ecosystem functioning. For example, Bardon et al. (2017) showed that *Fallopia* spp. complex (Asian knotweeds) produce high quantities of procyanidins that they were not considered to be self-toxic. The release of procyanidins by *Fallopia* spp. themselves induced a higher biomass allocation below ground and increases the lateral root production in *Fallopia* spp. and could also inhibit denitrification, thus improving nitrogen availability in nutrient-poor soils (Bardon et al., 2017). Thus, autoallelopathy of *L. hexapatala* could promote plant spread by increasing its competitive ability. Our first hypothesis on a positive effect of autoallelopathy of *L. hexapetala* on itself was validated.

Plant species may have developed resistance against allelochemicals from plants in the same habitat by co-evolution (Reigosa et al., 1999). Resistant species co-occurring with the donor plant could even benefit from the production of allelochemicals by the plant (Hilt et al., 2006). Many secondary metabolites, despite playing a primary role in defending the plant against pathogens or herbivores, can be considered to play secondary roles in plant-plant interactions, by which they nevertheless enhance the competitive potential of the plant (Reigosa et al., 1999). The putative allelochemicals released by the roots of *L. peploides* could directly favor the lateral growth of *L. hexapetala* or could also indirectly affect its development by modifying the chemical and physical properties of the soil and by regulating the soil microbial community (Walker et al., 2003) and by favoring the nutrient availability or altering pH (Blum et al., 1993). However, we have no evidence that the positive effect of root leachate of *L. peploides* on *L. hexapetala* traits results from a common past history and from co-evolution (it is possible that the two *Ludwigia* species introduced in France did not co-occur in their native range). Our study paved the way for future research about the allelopathic effects of *L. peploides* on *L. hexapetala* in both native and introduced ranges.

### Effects of Root and Leaf Leachates of *Mentha aquatica* and of *Myriophyllum aquaticum* on *L. Hexapetala* (Experiment 2)

No effect of root leachates was observed on the morphological traits of *L. hexapetala*. Parrot’s Feather *Myriophyllum aquaticum* has the same biological type with both aquatic and terrestrial forms as *L. hexapetala* and share the same niche. We suspected that to limit niche overlapping of both species, allelochemicals released by roots of *Myriophyllum aquaticum* do not affect the nutrient availability of soil and consequently they do not favor the nutrient acquisition and biomass of *L. hexapetala*. However, our results showed that the root leachate of *Myriophyllum aquaticum* had a positive effect on the flavonols in the leaves of *L. hexapetala.* Flavonoids are phenolic compounds that may be employed by plants as visual and olfactory attractants. Indeed, the leaf leachate of *Myriophyllum aquaticum* slightly stimulated the production of the lateral branches of *L. hexapetala.* The production of secondary metabolites by plants is determined by the genetic characteristics of the species producing them. Allelopathic plants may involve genetic changes within nearby growing plants. It may suggest that genotypes that are sensitive to allopathic chemicals have been removed from the gene pool, due to the continuous selection pressure of selective allelopathic chemicals, especially phenolic acids released by aquatic plants (Abbas et al., 2014). The two invasive species *Myriophyllum aquaticum* and *L. hexapetala* can coexist in field in their introduced range. The release of allelochemicals is also determined by the environmental conditions in which the plants are found (Reigosa et al., 2013). Variables such as temperature, humidity, and light intensity, added to the effects of the biota and the physicochemical structure of the soil, can affect not only the production of metabolites but also the chemical structure and degree of activity of substances released into the environment. Our hypothesis of “invasional meltdown” between the two sympatric species was validated.

In contrast, the hypothesis about a negative effect of the watermint *Mentha aquatica* leachates on the traits of *L. hexapetala* was invalidated. No effect of *Mentha aquatica* leachate on the *L. hexapetala* traits could be explained by the season and by the nature of the secondary compounds. Furthermore, the absence of effect of the watermint leachates could be due to the degradation of the allelochemicals after a short time. Secondary compounds can be degraded after they have been released into the soil; the half-life of allelochemicals varies from a few hours to a few months (Cheng and Cheng, 2015). This is mainly associated with the allelochemical concentration, soil type, and soil microbial population (Cheng and Cheng, 2015). Further studies are required to determine the concentration of these compounds in *L. hexapetala* soil and the stability of those compounds in the soil.

## Conclusions

The leachates of *L. hexapetala* favored its own synthesis of flavonols and its branching. This autoallelopathy could partly explain the water primrose invasiveness. The two invasive species *L. peploides* and *Myriophyllum aquaticum* stimulated the flavonols synthesis and the branching of the water primrose. These results suggested an “invasional meltdown.” Stimulation of the lateral growth and defense mechanisms by sympatric invasive species mediated by allelochemicals could potentially favor the persistence of *L. hexapetala* populations in invaded communities. The native *Mentha aquatica* leachate had no impact on the performance of the invasive *L. hexapetala*, showing no “biotic resistance.” These preliminary results must be taken carefully, while invasive plant growth was also determined by the interference between plants of the same or different species in the field. Deepening the understanding on plant-plant interactions has important implications for the management and the restoration of ecosystems that are both resistant and resilient to invasive species.

## Data Availability

All datasets generated for this study are included in the manuscript and/or the supplementary files.

## Author Contributions

GT and MT designed the experiment and conducted them. HR-P analyzed the data. GT wrote the manuscript with contributions from all the authors.

### Conflict of Interest Statement

The authors declare that the research was conducted in the absence of any commercial or financial relationships that could be construed as a potential conflict of interest.
